# Advanced Green Building Materials: From Resource Circularity to Low-Carbon Performance

**DOI:** 10.3390/ma19204283

**Published:** 2026-10-10

**Authors:** Michał Juszczyk, Karol Durczak, Agnieszka Sujak, Michał Pyzalski

**Affiliations:** 1 Faculty of Civil Engineering, Cracow University of Technology, Warszawska 24, 31-155 Cracow, Poland; 2 Faculty of Management, AGH University of Krakow, Al. Mickiewicza 30, 30-059 Cracow, Poland; karol.durczak@up.poznan.pl (K.D.); michal.pyzalski@agh.edu.pl (M.P.); 3 Faculty of Environmental and Mechanical Engineering, Poznan University of Life Sciences, Wojska Polskiego 50, 60-627 Poznan, Poland; agnieszka.sujak@up.poznan.pl; 4 Faculty of Materials Science and Ceramics, AGH University of Krakow, Al. Mickiewicza 30, 30-059 Cracow, Poland

The construction sector remains one of the most resource- and carbon-intensive components of the global economy. Buildings and construction are currently responsible for approximately 37% of global CO_2_ emissions and nearly 50% of global material extraction, demonstrating the scale of the environmental pressures associated with continued expansion of the built environment [1]. A substantial part of these impacts is linked to the extraction, processing, manufacture, and use of construction materials, including cement and concrete, metals, polymers, insulation products, and materials used in civil infrastructure [2,3,4]. As improvements in building energy efficiency progressively reduce operational emissions, greater attention is being directed towards embodied impacts and whole-life carbon, strengthening the need to consider environmental performance throughout material and building lifecycles [2,5]. At the same time, concerns over resource depletion, waste generation, and dependence on virgin raw materials are accelerating the transition from linear material consumption towards greater use of secondary resources, renewable feedstocks, industrial by-products, and other circular material strategies [2,3,4]. However, the substitution of conventional constituents with recycled, waste-derived, or bio-based alternatives cannot in itself be regarded as sufficient evidence of sustainability. Such materials must also exhibit suitable physicochemical characteristics, provide the required mechanical and functional performance, remain durable under relevant service conditions, and meet requirements related to processing, thermal behaviour, fire safety, environmental compatibility, and practical applicability [6,7]. The contemporary development of green construction materials is therefore moving beyond simple raw-material substitution towards integrated material systems in which resource circularity, lifecycle environmental performance, functionality, durability, and engineering applicability are considered simultaneously.

Against this background, the Special Issue Advanced Green Building Materials: Synthesis, Characterization and Sustainable Development Technologies brings together studies addressing the development, modification, characterization, and environmental assessment of materials intended for more sustainable applications across buildings and the wider built environment. The contributions can be grouped around several complementary research directions. These include the use of renewable and biomass-derived resources in construction products; the development of low-carbon cementitious systems and alternative supplementary cementitious materials; the valorisation of industrial, municipal, polymeric, and mining wastes in concrete, pavement, and backfilling applications; and the assessment of ecological functionality and lifecycle carbon performance. Collectively, these studies reflect a broader shift from the simple incorporation of alternative raw materials towards performance-oriented material design, in which processing conditions, microstructure, mechanical and functional properties, durability, environmental effects, and practical applicability are increasingly considered interdependent aspects of sustainable material development.

Importantly, the Special Issue resulted in eight published papers, demonstrating both the breadth of the topic and sustained interest in linking material innovation with environmental and engineering performance. Although the studies address different material groups and applications, they share a common emphasis on the valorisation of alternative resources, detailed material characterization, performance optimization, and assessment of practical applicability. The contributions range from bio-based and waste-derived construction products to modified cementitious materials, pavement materials, backfilling systems, and multifunctional ecological solutions, illustrating the diversity of pathways through which sustainability objectives can be incorporated into material design. At the same time, they demonstrate that environmental benefits need to be considered together with the technological requirements and functional performance of the resulting materials.

Renewable and biomass-derived resources represent one of the important pathways towards reducing dependence on virgin and fossil-based feedstocks in construction materials. Agricultural and agro-industrial residues are increasingly investigated as raw materials for engineered boards and other lignocellulosic products, where their valorisation may simultaneously reduce waste generation and pressure on conventional wood resources. In particular, binderless technologies offer an additional environmental advantage by exploiting the natural self-bonding capacity of lignocellulosic constituents and limiting the need for synthetic adhesives [8]. One contribution to this Special Issue follows this direction through the development of binderless fibreboards produced from agricultural residues and demonstrates how processing conditions influence their mechanical, structural, and moisture-related properties. A complementary approach is represented by the modification of polymeric insulation materials using biomass-derived fillers and ashes. Recent research on bio-based polyurethane systems indicates that renewable and waste-derived components can contribute to more sustainable insulation products, but their application requires simultaneous consideration of mechanical integrity, thermal performance, fire behaviour, emissions, and lifecycle impacts [7]. The results presented in this Special Issue reinforce this performance-oriented perspective, showing that the environmental potential of biomass and waste-derived constituents must be considered together with their influence on the functional properties and long-term applicability of the resulting construction materials.

Cementitious materials represent another major area in which environmental objectives are increasingly combined with material optimization and waste valorisation. Partial substitution of Portland clinker with supplementary cementitious materials (SCMs) remains one of the most established approaches to reducing the environmental impact of cement and concrete. However, changes in energy and industrial production systems may progressively limit the availability of conventional SCMs such as coal fly ash and ground granulated blast-furnace slag, increasing the need to identify and characterize alternative sources [6]. A growing range of industrial and mineral by-products is therefore being investigated, although their successful application depends not only on chemical composition but also on reactivity, processing requirements, consistency, durability, environmental compatibility, and compliance with relevant specifications [6]. This broader transition is also reflected in current industrial decarbonization strategies, which recognize greater use of supplementary materials, alternative raw materials, material efficiency, and the development of markets for low- and near-zero-emission cement and concrete as important elements of the pathway towards lower-carbon construction [9,10].

Several contributions to this Special Issue illustrate these directions at different levels of material development. Multi-component cementitious systems demonstrate the potential of combining several mineral additions and optimizing their proportions to balance mechanical performance with reductions in environmental impact. Other research focuses on lithium slag, a rapidly growing industrial by-product whose application as an SCM is attractive but complicated by substantial source-dependent variability in chemical composition and pozzolanic activity [11]. Processing and activation therefore become important tools for converting potentially reactive secondary resources into more reliable cementitious constituents. A similar need for careful material conditioning is evident in the utilization of municipal solid waste incineration fly ash. Although such residues offer opportunities for resource recovery, their composition may include chlorides, heavy metals, and other potentially harmful constituents, making pre-treatment, stabilization, and environmental safety essential considerations before their incorporation into cement-based materials [12]. Taken together, these studies show that the development of low-carbon binders is increasingly moving from straightforward cement replacement towards the deliberate engineering of secondary materials through characterization, activation, particle-size control, mixture optimization, and verification of both technical and environmental performance.

Circularity in construction materials also extends beyond building products and cementitious binders to infrastructure applications, where waste-derived constituents must satisfy demanding requirements related to processing, durability, and service performance. The reuse of polymeric wastes in asphalt materials is one example of this approach. Waste rubber and plastics can provide alternative modifier systems while simultaneously creating higher-value applications for difficult-to-manage waste streams. However, their effective use depends on compatibility between the modifier and asphalt matrix, storage stability, rheological behaviour, resistance to ageing, and performance across different temperature ranges [13]. One contribution to this Special Issue addresses these challenges through the combined use of reclaimed rubber, waste plastics, nanomaterials, and compatibilization techniques, illustrating how circularity increasingly relies on deliberate interface and material engineering rather than on the simple incorporation of waste constituents.

A related perspective is represented by the utilization of mining residues in cemented paste backfill systems. In such applications, the environmental benefit of redirecting tailings from surface disposal must be considered together with the requirements of material preparation, transport, and reliable placement. Particle-size distribution, solids concentration, yield stress, viscosity, sedimentation behaviour, and pipeline configuration strongly influence transport resistance, energy consumption, and operational stability [14]. The research presented in this Special Issue highlights the importance of these interactions by examining how aggregate grading affects slurry rheology and long-distance pipeline transport. This demonstrates that sustainable material utilization is inseparable from process optimization: the practical value of a secondary resource depends not only on its availability or composition but also on whether it can be processed, transported, and applied efficiently and reliably under realistic engineering conditions.

The development of sustainable construction materials is also increasingly associated with functions extending beyond conventional structural or thermal performance. Vegetated porous concrete provides an example of such multifunctional design, combining the mechanical requirements of an engineering material with porosity, water permeability, and conditions suitable for plant growth. Previous studies have demonstrated that the effectiveness of such systems depends on balancing sometimes competing requirements, particularly sufficient porosity for root development and water transport with adequate strength and stability for practical engineering applications [15]. One contribution to this Special Issue develops this perspective further by assessing porous vegetated concrete from a lifecycle standpoint and considering both the emissions associated with material production and construction and the potential carbon uptake occurring during service.

Such assessments illustrate both the opportunities and the methodological challenges associated with evaluating the climate performance of construction materials. Carbonation of cementitious materials can contribute to CO_2_ uptake over their service life and after demolition, while vegetation may provide an additional sequestration pathway. However, the magnitude of these benefits depends strongly on system boundaries, service-life assumptions, environmental exposure, end-of-life scenarios, and, importantly, the timing of emissions and subsequent carbon uptake. Recent research has shown that conventional accounting approaches may overestimate the climate benefit of cement carbonation when the delayed character of CO_2_ uptake and emissions associated with demolition and processing are not adequately considered [16]. Consequently, claims of net carbon-sink behaviour should be interpreted within clearly defined lifecycle boundaries and supported by sensitivity and uncertainty analyses. This broader perspective reinforces the need to evaluate green construction materials not only through their initial embodied impacts, but also through their long-term interaction with the environment and their performance throughout the complete lifecycle.

Despite the diversity of materials and applications represented in this Special Issue, a common methodological pattern can be identified across the contributions. Sustainable material development is increasingly based on the combined use of physicochemical characterization, mechanical and functional testing, microstructural analysis, rheological assessment, statistical optimization, numerical modelling, and lifecycle evaluation. These approaches are used not simply to determine whether an alternative constituent can be incorporated into a material, but to understand how its composition, particle characteristics, processing conditions, and interactions with other components influence the resulting structure and performance. This is particularly important for waste-derived and secondary materials, whose variability often requires additional processing, activation, compatibilization, or mixture optimization before reliable engineering performance can be achieved [6,13,14]. At the same time, the assessment of such materials increasingly extends beyond strength or other isolated technical parameters to include durability, thermal and fire behaviour, environmental compatibility, processability, and lifecycle effects [7,16]. The contributions to this Special Issue therefore illustrate a broader transition from proof-of-concept material substitution towards performance-engineered systems in which characterization and optimization provide the link between resource circularity, functional requirements, and environmental benefits.

Despite substantial progress in the development of lower-carbon, circular, and bio-based construction materials, several barriers continue to limit their broader implementation. Many proposed solutions remain at laboratory or pilot scale, while long-term field performance, durability under variable exposure conditions, consistency of secondary raw materials, and industrial-scale processing are still insufficiently documented. Practical adoption is also influenced by factors extending beyond material performance itself, including cost perceptions, allocation of responsibility, availability of reliable environmental data, established procurement practices, and the relatively risk-averse character of the construction sector [17]. More circular material systems may additionally require changes in supply-chain relationships, stakeholder roles, logistics, quality assurance, and business models, particularly when higher-value strategies such as reuse and closed-loop material circulation are pursued [18]. These challenges underline the importance of considering technological innovation together with the institutional and market conditions necessary for implementation.

Environmental assessment also remains an area requiring further methodological refinement. Lifecycle results can vary considerably depending on system boundaries, data sources, allocation procedures, transport assumptions, service-life scenarios, and end-of-life modelling, while conventional process-based approaches may omit relevant upstream impacts [19]. Greater methodological transparency, improved data quality, and more consistent assessment frameworks are therefore needed to enable meaningful comparison between conventional and alternative materials [19,20]. Similar attention should be given to potential trade-offs that are not always fully captured in material-development studies, including leaching and ecotoxicity, indoor emissions, fire performance, recyclability of newly created composites, availability and variability of waste streams, and the energy or environmental burden associated with pre-treatment and activation. Ultimately, the transition from promising laboratory formulations to widely applicable green construction materials will depend on the combined validation of technical performance, environmental benefits, economic feasibility, regulatory acceptance, and reliable supply at an appropriate scale [5,17,18].

Looking ahead, research on sustainable construction materials is likely to increasingly focus on the transition from laboratory-scale proof-of-concept studies towards material systems that can be validated under realistic production and service conditions. This may involve greater emphasis on pilot- and full-scale testing, long-term durability, standardized characterization and quality-control procedures for variable secondary raw materials, and the development of processing or activation routes that can be implemented at industrial scale [6,17,18]. At the same time, environmental assessment is likely to become more closely integrated with material design, with broader use of transparent lifecycle approaches, sensitivity and uncertainty analyses, and, where appropriate, consideration of economic performance and end-of-life scenarios [5,19,20]. Further progress may also depend on multi-objective optimization strategies that balance mechanical and functional performance with durability, carbon reduction, resource efficiency, cost, and recyclability. As the diversity of secondary and renewable resources continues to expand, greater attention may additionally be directed towards regional material availability, transport and supply-chain effects, environmental safety, and the compatibility of new material systems with existing standards and construction practices. Taken together, these trends suggest that the next stage in the development of green construction materials will increasingly be characterized by the integration of material science, environmental assessment, process engineering, and implementation-oriented research rather than by the isolated evaluation of alternative constituents.

The contributions presented in this Special Issue illustrate the broadening scope of sustainable construction-material research, from renewable and waste-derived feedstocks to low-carbon binders, circular infrastructure materials, and multifunctional systems with ecological and lifecycle benefits. Taken together, they show that the environmental value of alternative materials cannot be considered independently of their composition, processing requirements, mechanical and functional performance, durability, safety, and practical applicability. The development of green construction materials is therefore increasingly becoming a multidisciplinary task in which resource circularity, advanced characterization, performance optimization, and lifecycle assessment need to be considered together. Continued progress in this field will depend not only on identifying new secondary or renewable resources, but also on transforming them into reliable, scalable, and technically validated material systems capable of meeting the requirements of real construction and infrastructure applications. In this sense, the Special Issue also demonstrates the value of collaboration across complementary engineering disciplines in translating sustainable material concepts into technically validated and practically applicable solutions.

## Data Availability

No new data were created or analyzed in this study. Data sharing is not applicable to this article.
